# Safety and complications of continuation of aspirin therapy in patients undergoing robot-assisted laparoscopic simple prostatectomy

**DOI:** 10.1007/s11701-024-01946-2

**Published:** 2024-04-25

**Authors:** Sumit Saini, Parth U. Thakker, Rory Ritts, Timothy K. O’Rourke, Ashok K. Hemal

**Affiliations:** Department of Urology, Atrium Health Wake Forest Baptist Medical Center, 140 Charlois Boulevard, Winston-Salem, NC 27103 USA

**Keywords:** Benign prostatic hyperplasia, Large gland prostate, Robot-assisted simple prostatectomy, Aspirin, Safety, Surgical complications

## Abstract

To evaluate the safety and feasibility of continued perioperative aspirin at the time of robotic assisted simple prostatectomy (RASP). We performed a retrospective review of our IRB approved institutional database of patients who underwent RASP between 2013 and 2022. Comparative groups included patients taking aspirin in the perioperative period and those not taking aspirin pre-operatively. The primary outcome was any post-operative bleeding related complication using the modified Clavien–Dindo classification. Secondary outcomes included the identification of risk factors for increased blood loss in the entire study population, operative time, and blood transfusion requirement. 143 patients underwent RASP of which 55 (38.5%) patients continued perioperative aspirin therapy and 88 (61.5%) patients did not. Baseline demographics were similar between groups. Patients taking perioperative aspirin had a higher rate of hypertension (74.5% vs 58.0%, p = 0.04) and other cardiovascular disease (30.9% vs 11.4%, p = 0.007). Postoperative complications were similar between the groups (Clavien-Dindo ≥ 3; p = 0.43). Median blood loss (150 cc vs 150 cc, p = 0.38), percentage drop in hemoglobin (13.4 vs 13.2, p = 0.94) and blood transfusion rate (3.6 vs 1.1, p = 0.56) were also similar between groups. The median blood loss was 150 ml for the whole study population. On regression analysis, neither aspirin nor any other variable was associated with increased blood loss (> 150 ml). Aspirin can be safely continued perioperatively in patients undergoing RASP without any risk of bleeding related complications, blood loss, or increased transfusion rate.

## Introduction

Lower urinary tract symptoms (LUTS) secondary to benign prostatic hyperplasia (BPH) affects over 200 million men globally with up to 80% of men having the diagnosis of BPH by age 80 [1, 2]. While the initial management of LUTS secondary to BPH revolves around medical management, men failing or refusing to take medications may pursue surgical management. Endoscopic surgical therapies are typically utilized for small and medium size glands, however large glands (≥ 80 g) require alternative modalities [3, 4]. While holmium enucleation of the prostate (HoLEP) increases in popularity, there are notable barriers to entry for surgeons including equipment acquisition and a steep learning curve. Furthermore, many patients still require additional procedures after HoLEP. Thus, the most common surgical management of large and very large glands is the simple prostatectomy using either the open or robotic approach [5–8]. In either surgical approach, post-operative bleeding is a significant concern as the prostatic pedicles and dorsal venous complex are not controlled. While the robotic approach generally reduces blood loss, some studies have reported an estimated blood loss (EBL) of up to 600 cc [9]. This is particularly applicable to the aging male as many of these patients are on lifelong antiplatelet agents for cardiovascular or cerebrovascular disease. While anticoagulation is ubiquitously held in the pre-operative setting, holding antiplatelet agents is still debated as they are often critical in reducing post-operative morbidity and mortality.

Major adverse cardiovascular and cerebrovascular events (MACCE) are a considerable source of perioperative morbidity and mortality which occur in 1 in every 33 hospitalizations for non-cardiac surgery in the United States [10, 11]. Perioperative adverse cardiac events (PACE) including myocardial infarction, congestive heart failure, arrhythmias, acute pulmonary embolism, cardiac arrest, or stroke during the 30 day postoperative period are associated with long-term mortality [12]. Thus, prevention of these events is critical. Traditionally, aspirin is held in the pre- and post-operative setting for many urological procedures [13]. In recent years, the safety of continued perioperative aspirin for major urological operations including radical cystectomy and radical prostatectomy has been demonstrated, however, simple prostatectomy is an operation with a unique bleeding risk [14, 15]. Continued perioperative aspirin therapy may increase the risk of major bleeding as demonstrated previously by the Perioperative Ischemic Evaluation (POISE-2) trial [16]. In these scenarios, the risk of perioperative bleeding must be balanced with risk of PACE. Herein, we sought to evaluate the safety of continuing perioperative aspirin during robot-assisted laparoscopic simple prostatectomy (RASP).

## Methods

We retrospectively reviewed our prospectively maintained, IRB-approved database of all patients who underwent RASP by a single surgeon between 2013 and 2022. A total of 143 patients were identified. Data collection included patient demographics, medical co-morbidities, antiplatelet status, post-operative complications, preoperative and postoperative day 1 hemoglobin, estimated prostate volume based on preoperative imaging, history of prior BPH procedures, status of 5-alpha reductase inhibitors (5-ARI) and/or alpha antagonists, peri- and post-operative outcomes, and post-operative complications. The modified Clavien-Dindo classification was used to stratify post-operative complications with I–II considered minor and III-V considered major complications. Each patient had their Charlson Comorbidty Index (CCI) calculated based on factors at the time of surgery.Patients were divided into two groups, one group that continued aspirin therapy in the perioperative period and one group that was never on aspirin in the perioperative period. Additionally, all RASPs were performed using either the previously described Millin or Freyer techniques, and as such the prostatic pedicles were not controlled. No patients were given pre-procedure or post-operative deep vein thrombosis (DVT) pharmacologic prophylaxis. Mechanical DVT prophylaxis was used for all patients.

### Statistical analysis

Patient data was reported with median and interquartile ranges (IQR) for continuous variables or count and frequency for categorical variables. The cohort’s demographic, pathologic, complication, and antiplatelet status was compared with Wilcoxon rank-sum and Fisher’s exact for continuous and categorical data, respectively. All statistical testing was two-sided, and significance was defined as p < 0.05. Linear regression analysis was then utilized to determine which factors contributed to EBL. Statistical analyses were performed with Stata 14.0 software (2015, StataCorp Inc. College Station, Tx, USA).

## Results

We identified 143 patients undergoing RASP for BPH ≥ 80 g on pre-operative imaging, of which 55 (38.5%) patients continued perioperative aspirin therapy and 88 (61.5%) patients were not taking aspirin. Baseline and peri-operative characteristics of the entire cohort of patients receiving RASP are shown in Table 1. Demographics were similar between patients with continued aspirin and patients never on aspirin including age (70.9 vs 69.2 years, p = 0.15) and BMI (28.6 vs 27.6 kg/m^2^, p = 0.20). Rates of hypertension (74.5% vs 58.0%, p = 0.04) and cardiovascular disease (30.9% vs 11.4%, p = 0.007) were higher in the continued aspirin group. Preoperative PSA (10.3 vs 9.1 ng/dl, p = 0.50) and prostate volume (152.1 vs 141.3 g, p = 0.25) were also similar between the groups as were rates of prior prostate biopsy (63.6% vs 61.4%, p = 0.86), prior prostate surgery (16.4% vs 21.6%, p = 0.52), and patients on 5ARI (85.5% vs 85.2%, p = 0.48). Mean preoperative hemoglobin (13.8 vs 14.1 g/dL, p = 0.23), postoperative hemoglobin (11.9 vs 12.4 g/dL, p = 0.07), percent change in hemoglobin (13.4% vs 13.2%, p = 0.94), and blood transfusion rate (3.6 vs 1.1, p = 0.56) were not different between the two groups. Specimen weight (102.2 vs 99.9 g, p = 0.87), operative duration (163.6 vs 173.8 min, p = 0.07), and length of stay (2 vs 2 d, p = 0.62) were also no different between the groups. Patients continuing aspirin therapy did not have a higher transfusion rate (3.6% vs 1.1%, p = 0.56) or higher major postoperative complication rates (7.3% vs 3.4%; p = 0.43).

**Table 1:** Baseline patient characteristics and perioperative variables of patients undergoing robot-assisted laparoscopic simple prostatectomy comparing patients taking 81 mg aspirin and those never taking 81 mg aspirin

Variable	Aspirin (n = 55)	Non-aspirin (n = 88)	p-value
Baseline			
Age, mean (SD), years	70.9 (6.2)	69.2 (7.2)	0.15
BMI, mean (SD), kg/m^2^	28.6 (5.0)	27.6 (4.2)	0.20
Preoperative Hgb, mean (SD), g/dL	13.8 (1.7)	14.1 (1.5)	0.23
PSA, mean (SD), ng/dL	10.3 (8.7)	9.1 (6.8)	0.50
Preoperative prostate size, mean (SD), g	152.1 (71.5)	141.3 (63.2)	0.25
Preoperative prostate biopsy, n (%)	35 (63.6)	54 (61.4)	0.86
Prior prostate surgery for BPH, n (%)	9 (16.4)	19 (21.6)	0.52
Prior alpha blockers therapy, n (%)	47 (85.5)	75 (85.2)	1.00
Prior 5-ARI therapy, n (%)	36 (65.5)	52 (59.1)	0.48
Comorbidities			
DM, n (%)	13 (23.6)	19 (21.6)	0.83
HTN, n (%)	41 (74.5)	51 (58.0)	0.04
CAD, n (%)	17 (30.9)	10 (11.4)	0.007
CVD, n (%)	0 (0)	1 (1.1)	1.00
Simultaneous bladder surgery			
Bladder stone removal, n (%)	4 (7.3)	14 (15.9)	0.19
Bladder diverticulectomy, n (%)	2 (3.6)	4 (4.6)	1.00
Peri-operative			
Specimen weight, mean (SD), g	102.2 (56.6)	99.9 (54.0)	0.87
Operative time, mean (SD) min	163.6 (47.6)	173.8 (51.0)	0.07
EBL, median (IQR), cc	150 [100–200]	150 [100–200]	0.35
Post-operative Hgb, mean (SD), g/dL	11.9 (1.8)	12.4 (1.5)	0.09
Percentage drop in Hgb, mean (SD), %	13.4 (7.1)	13.2 (6.4)	0.94
Transfusion requirement rate, n (%)	2 (3.6)	1 (1.1)	0.56
Incidental prostate cancer final pathology, n (%)	10 (18.2)	13 (14.8)	0.64
Overall complication, n (%)	10 (18.2)	9 (10.2)	0.17
≥ Grade 3 [major] clavien-dindo complications, n (%)	4 (7.3)	3 (3.4)	0.43
Length of hospital stay, median (IQR), d	2 [1,2]	2 [1,2]	0.62

*BMI* body mass index, *PSA* prostate specific antigen, *BPH* benign prostatic hyperplasia, *5ARI* 5-alpha reductase inhibitor, *DM* diabetes mellitus, *HTN* hypertension, *CAD* coronary artery disease, *CVD* cerebrovascular disease, *EBL* estimated blood loss, *Hgb* hemoglobin

Univariate linear regression analysis was used to determine the impact of any given variable on operative blood loss (Table 2). None of the variables including antithrombotic medications (OR 0.63, 95% CI 0.33–1.20, p = 0.16), continued perioperative aspirin (OR 0.62, 95% CI 0.31–1.25, p = 0.18), prior prostate biopsy (OR 1.84, 95% CI 0.95–3.55, p = 0.07), prostate volume (OR 1.00, 95% CI 0.99–1.04, p = 0.23), prior BPH surgery (OR 0.68, 95% CI 0.30–1.55, p = 0.36), prior alpha blocker therapy (OR 1.14, 95% CI 0.48–2.73, p = 0.76), prior 5ARI therapy (OR 0.98, 95% CI 0.50–1.88, p = 0.95), operative time (OR 1.00, 95% CI 0.99–1.01, p = 0.40), and specimen weight (OR 1.02, 95% CI 0.84–1.23, p = 0.83) were found to be associated with increased blood loss. Additionally, none of the comorbidities assessed including Diabetes Mellitus (OR 1.24, 95% CI 0.58–2.67, p = 0.70), hypertension (OR 1.16, 95% CI 0.60–2.25, p = 0.73), cardiovascular disease (OR 1.26, 95% CI 0.59–2.66, p = 0.57), or cerebrovascular disease (OR 1.34, 95% CI 0.08–1.88, p = 0.87) were associated with increased blood loss. Finally, no increased blood loss was associated with simultaneous bladder surgery including bladder stone removal (OR 1.23, 95% CI 0.47–3.23, p = 0.66) or diverticulectomy (OR 0.66, 95% CI 0.11–3.71, p = 0.63).

**Table 2:** Univariate analysis of factors related to blood loss during robot-assisted laparoscopic simple prostatectomy

Variable	OR	95% CI	p-value
Antithrombotic medications	0.63	0.33–1.20	0.16
Continued aspirin (peri-operatively)	0.62	0.31–1.25	0.18
Prior prostate biopsy	1.84	0.95–3.55	0.07
Pre-operative prostate volume	1.00	0.99–1.04	0.23
Prior surgery for BPH	0.68	0.30–1.55	0.36
Comorbidities			
Diabetes mellitus	1.24	0.58–2.67	0.70
Hypertension	1.16	0.60–2.25	0.73
Cardiovascular disease	1.26	0.59–2.66	0.57
Cerebrovascular disease	1.34	0.08–21.86	0.87
Prior alpha blocker therapy	1.14	0.48–2.73	0.76
Prior 5ARI therapy	0.98	0.50–1.88	0.95
Simultaneous bladder surgery			
Bladder stone removal	1.23	0.47–3.23	0.66
Diverticulectomy	0.66	0.11–3.71	0.63
Operative time	1.00	0.99–1.01	0.40
Specimen weight	1.02	0.84–1.23	0.83

*BPH* benign prostatic hyperplasia, *5ARI* 5-alpha reductase inhibitor

Patients who underwent RASP in the first 5 years of the study period were compared to patients who underwent RASP in the latter 5 years (Table 3). No differences were noted in terms of age (69.1 vs 70.6, p = 0.12), BMI (27.2 vs 28.5, p = 0.07), CCI (3.9 vs 3.8, p = 0.43), ASA status (2.6 vs 2.7, p = 0.11), operative time (171 vs 168, p = 0.39), EBL (208 vs 184, p = 0.19), specimen weight (108 vs 99, p = 0.2), percentage of patients with continued ASA 81 mg use (39% vs 33%, p = 0.72), or rate of any Clavien-Dindo complication (15% vs 14%, p = 0.85).

**Table 3:** Baseline patient characteristics and perioperative variables of patients undergoing robot-assisted laparoscopic simple prostatectomy during the first 5 years versus the second 5 years of the study period

Variable	First 5 years (n = 39)	Second 5 years (n = 104)	p-value
Age, mean (SD), years	69.1 (6.8)	70.6 (6.8)	0.12
BMI, mean (SD), kg/m^2^	27.2 (4.5)	28.5 (4.7)	0.07
CCI, mean (SD)	3.9 (2)	3.8 (2)	0.43
ASA, mean (SD)	2.6 (0.6)	2.7 (0.6)	0.11
EBL, median (IQR), cc	208 (108)	184 (159)	0.19
Operative time, mean (SD), min	171 (51)	168 (49)	0.39
Specimen weight, mean (SD), g	108 (52)	99 (56)	0.2
Taking ASA 81 mg, n (%)	15 (39)	34 (33)	0.72
Any clavien–dindo complication, n (%)	6 (15)	15 (14)	0.85

*BMI* body mass index, *CCI* Charlson comorbidity index, *ASA* Association of Anesthesiologist Score, *EBL* estimated blood loss, *ASA* aspirin

## Discussion

Aspirin remains the most widely prescribed medication for the primary and secondary prevention of cardiovascular events. In this retrospective analysis of patients undergoing RASP, we demonstrated the safety of continued perioperative aspirin therapy with no increased risk of post-operative bleeding, major complication rates, or transfusion requirement. To the best of our knowledge, this is the first study highlighting such outcomes in patients undergoing RASP.

Simple prostatectomy is the recommended surgical option in patients with symptomatic large and very large volume prostates. This disease process primarily afflicts the elderly male, many of whom are on long-term anticoagulation or antiplatelet therapy. The decision regarding aspirin discontinuation in the perioperative period is contingent on both patient-related and procedure-related factors. This should be highly individualized and multidisciplinary after thoroughly assessing the benefits and risks involved [17]. A meta-analysis noted a three-fold higher risk of MACE in patients who are non-compliant with or holding aspirin therapy [18]. In contrast, a large multinational, multicenter randomized trial (POISE-2) noted no benefit of aspirin continuation in terms of major adverse cardiovascular events. Interestingly, the risk of major bleeding was found to be higher in those with continued aspirin therapy. Thus, the decision regarding discontinuation of aspirin should be considered with due diligence.

With increasing familiarity and advances in robotic surgical systems, along with its expansion to a variety of disease processes, RASP is gradually replacing the open approach due to improved peri-operative and functional outcomes [19–21]. Previously published studies comparing the robotic and open approaches have highlighted favorable transfusion rates, catheterization time, major complication rates, and hospital stays with comparable functional outcomes for RASP [5–8]. Benorroche et. al. demonstrated superior hemostasis with the robotic approach with a median blood loss of 200 cc vs 400 cc for the open approach. Likewise, Sorokin et. al. found a decreased blood loss with RASP compared to the open approach (339 cc vs. 587 cc) [7, 22]. In our analysis, the median blood loss was found to be 150 cc for the entire population, which was similar between the two groups (150 cc vs 150 cc, p = 0.38). Likewise, continued perioperative aspirin therapy was not associated with increased transfusion rate (2% vs 1%, p = 0.53). One may hypothesize that complication rates, blood loss, and operative time may be longer during the early phase of our study, however, our analysis revealed no significant differences in the first and second 5 years of the study period.

The overall and major complication rates in our study were 14.4% and 5.7%, respectively, and we found no increase in operative blood loss (p = 0.35) or decrease in hemoglobin (p = 0.94) with the continuation of perioperative aspirin during RASP. These findings are similar to those noted by Carniero et al. who analyzed the safety of continued aspirin therapy in patients undergoing robotic radical prostatectomy [15]. On regression analysis, continued aspirin use was not found to be associated with an increase in the blood loss, although the odds of increased blood loss were approaching clinical significance (OR 1.84; 95% CI 0.95 – 3.55; p = 0.07) in patients with prior prostate biopsy. These novel findings support continuing aspirin perioperatively during RASP when clinically indicated.

While this study highlights the safety of perioperative aspirin therapy in RASP, it is not without limitations. The retrospective design and lack of a cohort taking aspirin for secondary prevention preclude the analysis of cardiovascular events between the groups. Second, complication rates after the robotic approach to simple prostatectomy are low in general, and a larger sample size may have uncovered these associations. Despite these limitations, our study indicates that continued perioperative aspirin administration in patients undergoing RASP is not associated with increased risk of bleeding, blood transfusions, or increased complication rates.

## Conclusions

Continued administration of perioperative aspirin appears to be safe in patients undergoing robot-assisted laparoscopic simple prostatectomy without an increase in the risk of bleeding, transfusion requirements, or post-operative complications.

## Data Availability

The data that support the findings of this study are available on request from the senior author, A.K.H. The data are not publicly available due to potential confidential information that could compromise the privacy of research participants.
